# Health worker and policy-maker perspectives on use of intramuscular artesunate for pre-referral and definitive treatment of severe malaria at health posts in Ethiopia

**DOI:** 10.1186/s12936-016-1561-6

**Published:** 2016-10-18

**Authors:** Takele Kefyalew, Zelalem Kebede, Dawit Getachew, David Mukanga, Tessema Awano, Agonafer Tekalegne, Esey Batisso, Wasihun Edossa, Emebet Mekonnen, James Tibenderana, Ebenezer Sheshi Baba, Constance Shumba, Joaniter I. Nankabirwa, Prudence Hamade

**Affiliations:** 1 Malaria Consortium Ethiopia, Addis Ababa, Ethiopia; 2 Science and Health Impact Group, Kampala, Uganda; 3 Oromia Regional Health Bureau, Addis Ababa, Ethiopia; 4 SNNPR Health Bureau, Hawassa, Ethiopia; 5 Malaria Consortium Uganda, Kampala, Uganda; 6 Makerere University College of Health Sciences, Kampala, Uganda; 7 Malaria Consortium UK, London, UK

**Keywords:** Severe malaria, Intramuscular artesunate, Perspectives, Intra-rectal artesunate, Health post, Health extension worker

## Abstract

**Background:**

The World Health Organization (WHO) recommends injectable artesunate given either intravenously or by the intramuscular route for definitive treatment for severe malaria and recommends a single intramuscular dose of intramuscular artesunate or intramuscular artemether or intramuscular quinine, in that order of preference as pre-referral treatment when definitive treatment is not possible. Where intramuscular injections are not available, children under 6 years may be administered a single dose of rectal artesunate. Although the current malaria treatment guidelines in Ethiopia recommend intra-rectal artesunate or alternatively intramuscular artemether or intramuscular quinine as pre-referral treatment for severe malaria at the health posts, there are currently no WHO prequalified suppliers of intra-rectal artesunate and when available, its use is limited to children under 6 years of age leaving a gap for the older age groups. Intramuscular artesunate is not part of the drugs recommended for pre-referral treatment in Ethiopia. This study assessed the perspectives of health workers, and policy-makers on the use of intramuscular artesunate as a pre-referral and definitive treatment for severe malaria at the health post level.

**Methods:**

In-depth interviews were held with 101 individuals including health workers, malaria focal persons, and Regional Health Bureaus from Oromia and southern nations, nationalities, and peoples’ region, as well as participants from the Federal Ministry of Health and development partners. An interview guide was used in the data collection and thematic content analysis was employed for analysis.

**Results:**

Key findings from this study are: (1) provision of intramuscular artesunate as pre-referral and definitive treatment for severe malaria at health posts could be lifesaving; (2) with adequate training, and provision of facilities including beds, health posts can provide definitive treatment for severe malaria using intramuscular artesunate where referral is delayed or not possible; (3) health workers at health centres and hospitals frequently use the intravenous route because it allows for co-administration of other drugs, but they find the intramuscular route easier to use at the health post level; (4) the reasons commonly cited against the management of severe malaria using intramuscular artesunate at health post level were: lack of capacity to manage complications and fear of irrational drug use; (5) use of intramuscular artesunate at health post level will require evidence on safety and feasibility before policy shift.

**Conclusion:**

From the perspective of health workers, use of intramuscular artesunate as pre-referral treatment of severe malaria cases at the health post is possible but dependent on training and availability of skilled workers. Use of intramuscular artesunate as definitive treatment at health posts was not supported, however, operational research to establish its feasibility, safety and efficacy was recommended to guide any implementation of such an intervention.

## Background

Malaria is endemic throughout most of the tropics, with approximately 214 million cases and 438,000 deaths reported in 2015 alone [1]. About 88 % of cases and 90 % of deaths occur in the World Health Organization (WHO) African region, and 70 % of deaths are among children under 5 years of age [1]. In endemic areas, young children and pregnant women are at high risk for severe malaria [2, 3].

Severe malaria is a life threatening medical emergency that requires prompt and effective treatment to prevent death [1, 4]. Death from severe malaria often occurs within hours of admission to health facilities due to problems with patients accessing treatment in a timely manner. It is essential that therapeutic concentrations of a highly effective anti-malarial drug are achieved as soon as possible, and that there is access to curative treatments as close as possible to where the patient lives. Compared to most other malaria-endemic countries in Africa, malaria prevalence is relatively low in Ethiopia with the parasite prevalence estimated at 0.5 % in the recently concluded malaria indicator survey [5]. Ethiopia was ranked 10th in terms of estimated incidence of malaria in East Africa in 2015 [1]. However, despite a low prevalence, malaria is a major public health problem and is one of the four leading cause of illness in children under 5 years of age in Ethiopia [6].

The current WHO recommendation is to give a parenteral antimalarial drug to treat severe malaria for a minimum of 24 h irrespective of the patient’s ability to tolerate oral medication, before giving the oral follow-up artemisinin-based combination therapy (ACT) [7]. The risk of death in severe malaria is greatest in the first 24 h, yet the transit time between referral and arrival at health facilities with access to parenteral treatments is usually long. Mortality in untreated severe malaria cases approaches 100 % [7], and especially in areas of low, unstable malaria transmission [8]. Ethiopia has unstable malaria transmission and, therefore, epidemics are serious public health emergencies.

Referral of severely ill patients is faced with many challenges including lack of transport, long distance to health facilities, lack of funds for transport, distances to be covered, lack of referral slip, poor communication and poor road networks [9]. WHO recommends that where complete treatment of severe malaria is not possible patients be given single dose of the recommended parenteral treatment before referral [7, 10]. It recommends that where complete treatment of severe malaria is not possible but injections are available, adults and children should be administered as referral treatment, a single intramuscular dose of artesunate or intramuscular artemether or intramuscular quinine, in that order of preference before referring to an appropriate higher health facility. Where intramuscular injections are not available, children under 6 years may be administered a single dose of rectal artesunate [7].

In developing countries, pre-referral care for severe illnesses such as severe malaria face major challenges due to frequent stock-outs or shortages of essential supplies, and poorly organized emergency treatment [11, 12]. The basic components of severe malaria case management are lacking in most health facilities including oxygen and blood transfusion [13].

**Fig. 1:**
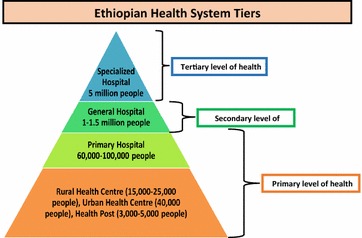
Different tiers of the Ethiopia health system and the approximate population they cover

The Ethiopian government is making efforts to increase access to care for the entire population. However, in many areas access to qualified specialist care is still limited. The Ethiopian health service is restructured into a three tier system: (1) primary health care unit consisting of a primary hospitals, health centres and health posts, (2) general hospitals, and (3) specialized hospitals (Fig. 1). The health posts are managed by two health extension workers who are recruited among high school graduates in local communities, and undergo a 1-year training program to deliver a package of preventive and basic curative services that fall under four main components: hygiene and environmental sanitation; family health services; disease prevention and control; and health education and communication. The health centres are staffed by around 20 professionals and provide preventive, curative, inpatient and ambulatory services, treatment of common psychiatric disorders, and dental services. Primary hospitals are staffed by around 53 professionals and provide preventive, curative, inpatient and ambulatory services, and emergency surgical services, including caesarean section and blood transfusion. They also serve as referral centres for health centres and practical training centres for nurses and paramedical health professionals. General hospitals are staffed by around 234 persons and provide inpatient and ambulatory services. They are also referral centres for primary hospitals and training centres for health officers, nurses, emergency surgeons and other categories of health workers. The specialized hospital is staffed by around 440 professionals and serves as a referral centre for the general hospitals and provides inpatient services.

The health workforce density in Ethiopia increased from 0.84 to 1.3 per 1000 population between 2008 and 2013 indicating improvement in supply and availability of health workers. However, the doctor, health officer, nurse and midwife to population ratio is 0.7 per 1000 population, far behind the minimum threshold of 2.3 doctor, nurse and midwife to 1000 population ratio required to ensure high coverage with essential health interventions. Nevertheless, the health extension programme has over 38,000 health extension workers working in more than 16,000 health posts thereby reaching households with essential health packages [14, 15].

In the national strategic plan 2014–2020, Ethiopia has a target of 100 % access to effective and affordable malaria treatment and to achieve near zero malaria deaths by 2020 [6]. This requires improving diagnosis of malaria cases using microscopy or using multi-species rapid diagnostic tests (RDTs), and providing prompt and effective malaria case management at all health facilities level in the country. Most malaria cases are first seen and treated at health posts and health centre level. According to the national malaria treatment guidelines, patients arriving at a health post with severe malaria are given intrarectal artesunate or intramuscular artemether when intrarectal artesunate is unavailable [16]. Unfortunately, there are no prequalified manufacturers for rectal artesunate and its use is only recommended for children under 6 years [17]. WHO recommends intramuscular artesunate for pre-referral treatment [7], but this is not currently included in the national policy guidelines in Ethiopia [16] to be delivered by health extension workers in Ethiopia.

Intravenous injections require cannulation and may be beyond the level of skills found at health posts; however, intramuscular injections are administered at this level. Studies have shown that intramuscular artesunate is rapidly absorbed when used for managing severe malaria in contrast to artemether which is oil based and absorption may be delayed especially in shocked patients [18, 19]. Intramuscular and intravenous artesunate have also been reported to be equally effective in adults [20] and children [21].

In hard to reach areas where referral of severe malaria cases is not possible or delayed, the only option left to patients and caregivers is to return home and use whatever is available within their community, at great danger to lives [22]. For these settings could health posts that are closer to communities, and where health extension workers have been properly trained and equipped provide pre-referral treatment for severe malaria using intramuscular artesunate as recommended by WHO? Could they also provide definitive treatment (defined as *the treatment plan for a disease that has been chosen as the best for a patient after all other choices have been considered*) where referral is delayed or not possible, given that many already provide intramuscular treatments for other conditions? This study sought to explore the perspectives of health workers, and policy makers on the use of intramuscular artesunate as a pre-referral and definitive treatment for severe malaria at health post level in Ethiopia. This will provide valuable information on the policy considerations, possible challenges to such a policy, and mitigation measures.

## Methods

### Study area

The study was conducted in two regions of Ethiopia, southern nations, nationalities, and peoples’ region (SNNPR), and Oromia from January to March, 2015. Participants were identified from health facilities serving nine malarious areas of the regions, Zonal offices, Regional Health Bureaus, Federal Ministry of Health and partners.

Oromia is the most populous region in Ethiopia with a total population of 32 million. SNNPR is the 3rd largest regional state with a total population of 18 million [6]. SNNPR and Oromia regions have the highest malaria burden in Ethiopia [23].

The major health problems of SNNPR and Oromia remain largely preventable communicable diseases and nutritional disorders. The health system priorities are health service delivery at household, community and facility level to improve maternal, neonatal, child, adolescent and youth health, nutrition, hygiene and environmental health, and to reduce/combat HIV/AIDS, tuberculosis and malaria and other communicable and non-communicable diseases [15].

Access to care remains a problem especially in rural areas where the population is characterized by poverty and poor health indicators. The Ethiopian government has endeavored to improve access to care by providing a community based service provided by health extension workers who after a year’s training provide comprehensive preventive and curative services to these remote populations and by equipping health centres with ambulances to provide care for obstetric and other emergencies but referral from health post could be delayed due to poor infrastructure or other emergencies. The Ethiopia health care tiers are presented in Fig. 1.

### Study design

This was a qualitative exploratory study that used in-depth interviews to collect data on the perspectives of health workers, and policy-makers on the use of intramuscular artesunate as a pre-referral and definitive treatment for severe malaria at health posts.

### Sample size and sampling technique

A total of 101 respondents were interviewed. A list of all zones from the two regions regarded high burden by the Federal Ministry of Health was used and from this list nine zones with the highest burden were selected. The selected zones were the following: East Shoa, South west Shoa and Jima zone from Oromia region, and Gomgofa, Silti, Kembata Tembaro, Halaba, Wolayita and Hadiya zones from SNNPR. A zone is an administrative area below the region, and includes several districts or *woredas*.

Thirty health facilities from each of the two states (Oromia and SNNPR) were purposively selected to be included in the study if they had large numbers of severe malaria cases reported (according to the routine health facility data reports) and if were easily accessible by the study team.

### Study population

A maximum of two health workers, one health worker involved in management of malaria on the day of the interview and one manager in the selected health facilities were included in the study. In Oromia state, given the busy schedules of the health workers, it was impossible to interview more than one staff at the selected facilities, and two of the selected facilities were not open on the survey day. In SNNPR, all selected facilities were functional and two health workers were interviewed at each of the facilities. At two of the facilities in SNNPR, the team was able to interview both the severe malaria ward manager and well as the health facility manager. In addition, the malaria focal persons from the Zonal health departments, Regional Health Bureau, Federal Ministry of Health, and development partners were also included in the study. The number of respondents interviewed within the different categories are presented in Table 1.

**Table 1:** Respondents selected by category and region

Category	Region		Total
	SNNPR	Oromia	
Health post staff	30	13	43
Health centre staff	25	10	35
District, zonal and regional staff	4	3	7
Hospital staff	7	5	12
FMOH	N/A	N/A	1
Partners	N/A	N/A	3
Total	66	31	101

### Data collection

An interview guide was used to collect data from respondents. In-depth interviews were conducted by twenty research assistants with research experience and trained in data collection specifically for this study. The interviews were conducted in the local languages. The research assistants working in pairs approached the managers and asked permission to conduct the study in the health facilities and for guidance on the health workers to be interviewed. Informed consent was then sought from the health workers to participate in the study. Appointments were made for interviews with all other participants at zonal, regional, Federal Ministry of Health and development partners. The research assistants took notes and audio recordings during the interviews. Supervision and guidance to the field teams was provided by two co-investigators with experience conducting qualitative research. Daily supervision of interviewers and checking of completed interviews was done to ensure collection of accurate and complete data.

### Data analysis

Audio-recorded data were transcribed and then translated from the local languages to Amharic and then into English. Senior researchers read the transcripts and identified emerging themes, and codes. The transcripts were then coded using Atlas.ti7 (Atlas.ti GmbH, Berlin) during the coding process; more codes were identified and discussed by the research team. These codes were merged into categories and then into themes reflecting the study objectives and other emerging issues. Thematic content analysis was employed.

## Results

### Characteristics of the study population

A total of 101 respondents were interviewed (Table 1). Of these, 66 (66.7 %) and 31 (31.3 %) were from SNNPR and Oromia regions respectively. The majority (73/101; 72 %) of the participants worked at various health facilities providing treatment and care for severe malaria cases. Other participants were from Zonal and Regional health departments or district health offices (7), Health Bureau and Federal Ministry of Health (1) and partner institutions (3). 63 % of the participants were female, while the average age of participants was 28 years (standard deviation 6.3). 65 % of the participants held either a degree in public health or nursing; 27 % held a certificate or diploma; while 8 % held a Masters’ degree. The average duration of service of the respondents in the health sector was 4.8 years.

### Knowledge and views of participants on current treatment guidelines

Several respondents knew that the current recommended treatment for uncomplicated malaria is artemether–lumefantrine for *Plasmodium falciparum* and chloroquine for *Plasmodium vivax.* Many participants knew that as injectable artesunate is the drug of choice for treatment of severe malaria.

On the other hand, a few respondents did not know the drug of choice for treatment of severe malaria cases. This lack of knowledge was found at both health centres and among health extension workers.

*Quinine is a drug of choice for treatment of severe malaria. Injectable artesunate is given when a patient is not responding to quinine* (Health centre staff, Oromia region).

Information on use of rectal artesunate as pre-referral treatment at community/health post level was limited with the many of the respondents indicating that patients with severe malaria should be treated in hospital and health centres.

### Health workers practices around the use of intramuscular or intravenous artesunate

Both respondents from health centres and hospitals said that injectable artesunate is commonly administered in their health facility and the intravenous route was the most commonly cited route for the administration of treatment for severe malaria. However, one participant from one health centre said that ‘Intramuscular injection is a common route in her facility.’

Participants said that the ‘intramuscular route is an alternative route for administration of injectable artesunate’. The most common sources of information stated by participants on intramuscular route of administration of injectable artesunate in descending order were: trainings, malaria case management guidelines, and fellow health workers.

Ninety-nine percent of the participants responded erroneously that ‘Gluteal muscle is the site of administration for adult patients’ and only a couple of respondents correctly responded that ‘the deltoid and/or ventrolateral muscles are preferred site for intramuscular artesunate.’

### Benefits of using intramuscular artesunate

Although the respondents especially those working at hospitals and health centres stated that they use intravenous artesunate, they noted that the intramuscular route is easier to administer.

*Yes, intravenous needs cannula, but* [for] *intramuscular there is no need for cannula, simply dilute and give the dose* (Health Centre staff, SNNPR).

*The only challenges could be in getting the vein entry at one single shot* [for intravenous] (Health Centre staff, SNNPR).

However, some participants thought intravenous artesunate is faster acting than intramuscular. Another benefit identified was that less intense training is required for health workers before they can rightly administer intramuscular artesunate.

*Intramuscular artesunate is not used often because intramuscular action is slow when compared to the intravenous and also intramuscular artesunate causes pain* (Health Centre staff, SNNPR).

### Possible barriers to using intramuscular injection at post referral facilities

Participants stated that no challenges should be expected as intramuscular artesunate is already included in the treatment guideline at the health centre and hospital level but the confidence of using the intravenous route might affect the use of intramuscular injection.

…*the prevailing skill and knowledge of health workers who have been using intravenous quinine for treatment of severe malaria may negatively influence the use of intramuscular artesunate*. (Medical Doctor at hospital, Oromia region).

Some participants also anticipated low usage of intramuscular artesunate for treatment of severe malaria because intravenous medication facilitates the administration of other intravenous drugs.

*If the patient was on open intravenous cannula for another purpose, we also use the intravenous route not to add another burden of intramuscular administration on the patient* (District malaria focal person, SNNPR).

*As I said, based on* [the] *patient’s condition we choose the route. If* [the patient is] *critical and need[s] additional management, we prefer intravenous route. Otherwise, Intramuscular injections are better* (Medical Doctor at District Hospital, SNNPR).

The participants expressed the opinion that quinine might continue to be used because of stock outs of injectable artesunate due to poor drug and supply management affecting the use of intramuscular artesunate. The health workers stated that client preference of intravenous route was also a barrier to the use of intramuscular injections.

*Those clients who are admitted are not happy with intramuscular artesunate administration, so for patient satisfaction purpose we use intravenous artesunate*. (District malaria focal person, Oromia).

### Practicality of health extension workers providing care for severe malaria using intramuscular artesunate as definitive as well as pre-referral treatment

Participants said that lower health facilities especially health posts are not in a position to treat severe malaria using intramuscular artesunate because of the national policy doesn’t support it, extension workers have limited knowledge and skills of managing severe malaria complications, there is limited availability of suitable facilities at the health post.

*As lower level health facilities do not have inpatient beds and facilities; they cannot manage severe malaria patients. Since severe malaria is a complicated medical emergency, severe malaria cases should be referred to hospitals.* (District hospital staff, SNNPR).

*…it is difficult for them because there may be other comorbidities which needs treatments like infusions. So admitting the patient, it may be difficult.* (Zonal malaria focal person, Oromia region).

Respondents suggested that with adequate training, and provision of beds and other facilities health posts would be able to manage severe malaria with intramuscular artesunate if referral was not possible and would also be able to provide pre-referral treatment with intramuscular artesunate.

*It is possible for me to treat because I already give vaccination and depo* [family planning injection] *which I did not know how to give before my education and training* (health extension worker, Oromia region).

### Benefits and risks of managing severe malaria with intramuscular artesunate at lower level facilities

Participants were concerned that health extension workers would not be able to manage complications often associated with severe malaria.

*If health extension workers manage patients with severe malaria, case fatalities of malaria will increase; intramuscular artesunate should only be provided as a pre*-*referral treatment at health post level* (Participant, partner organization).

A few participants raised the potential risk of drug resistance due to irrational use of intramuscular artesunate, as it could be used for uncomplicated malaria.

*Community health care workers cannot administer intramuscular artesunate for the treatment of severe malaria because of their educational status. Parasite may adapt* [to] *the drug if not administered properly.* (Health worker, Oromia region).

Some respondents thought that given the accessibility of health posts to the community would empower them to manage severe malaria with intramuscular artesunate and this would save lives if referral was not possible or if there were delays in getting care.

*Health posts are near to the community which does not require transportation. If a patient gets sick at night, health posts are the nearest health facility to the community, therefore allowing health post to treat severe malaria cases using intramuscular artesunate is advisable* (Health centre staff, Oromia region).

*I am very happy, because this particular area is highly malaria endemic, if patient go to Healthcentre, he/she may not get service rapidly because* [the] *Health centre serve*[s] *a lot of population, but we are serving small population so* [it is] *easy for us to serve kebele* [village] *population* (health extension worker, SNNPR).

### Policy implications

Participants involved in policy advice reported that intramuscular artesunate is already included in the treatment guideline for use at hospital and health centre level. Some of participants indicated that health extension workers can use intramuscular artesunate as pre-referral treatment, but not definitive treatment of severe malaria once trained. Malaria experts who formulate policy said it is possible to revise the national treatment guidelines to include intramuscular artesunate as pre-referral treatment at community level once evidence supports its feasibility.

## Discussion

### Key findings

The key findings from this study are: (1) majority of participants knew the contents of the current treatment guidelines for uncomplicated malaria and for severe malaria at hospital level and health centre level but many did not know about the use of rectal artesunate and intramuscular artesunate for pre-referral treatment; (2) provision of intramuscular artesunate as pre-referral and definitive treatment for severe malaria at health posts could be lifesaving in situations where referral is not possible; (3) with adequate training, and provision of facilities including beds, health posts can provide injectable artesunate as pre-referral medication, however, there was hesitation of using this as definitive treatment for severe malaria (4) health workers in health centres and hospitals prefer the intravenous route because it can be used for co-administration of other drugs but suggest that in the case of health extension workers the intramuscular route is easier; (5) the most common reasons cited for not supporting the definitive management of severe malaria using intramuscular artesunate at community level were; lack of capacity to manage complications of severe malaria cases at lower level health facilities and Fear of irrational use of the drug. (6) Intramuscular artesunate is already included in the national policy for health centres and hospitals, but pre-referral intramuscular artesunate at health post level will require evidence on safety and feasibility before policy shift.

**Fig. 2:**
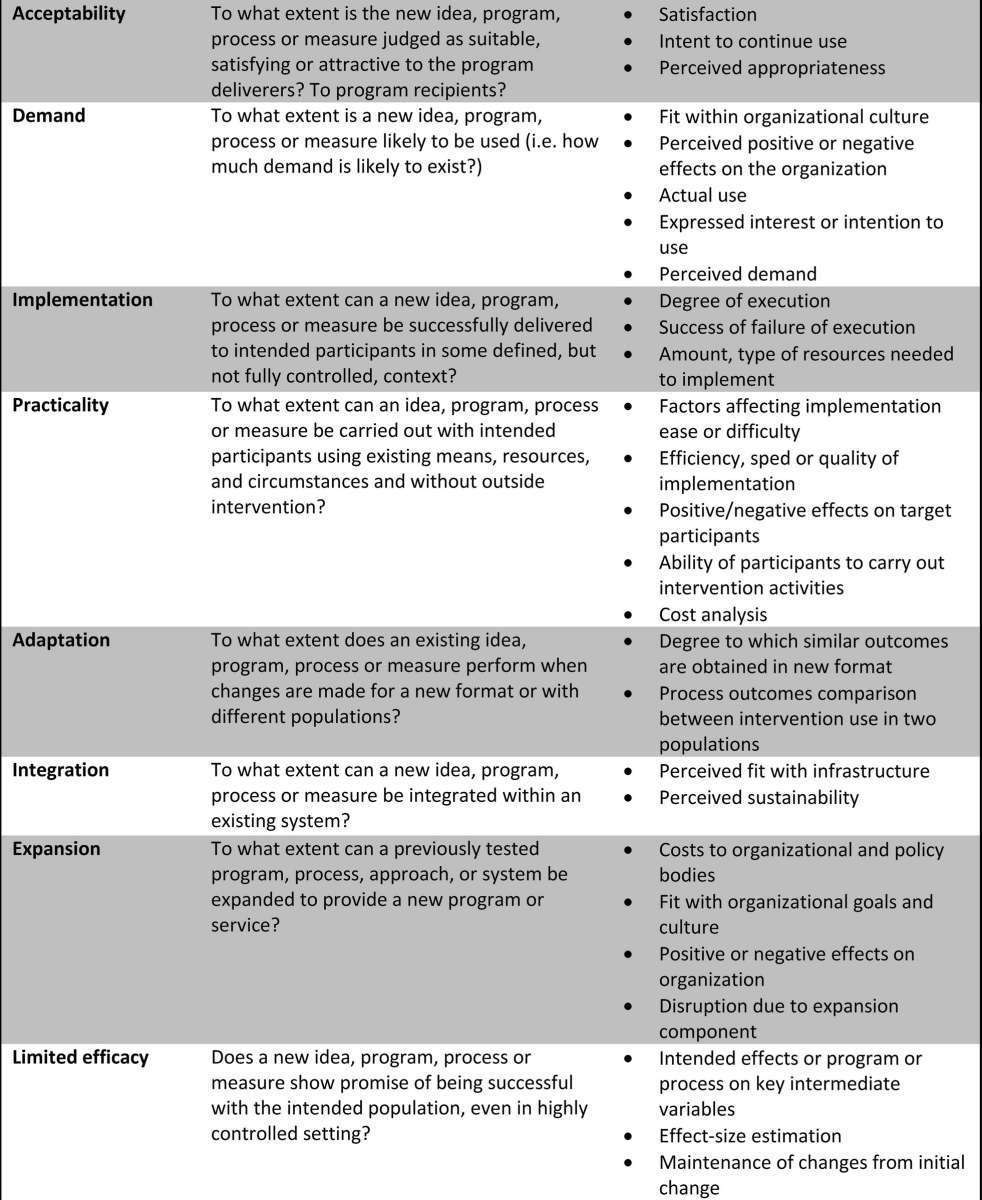
Key areas of focus for feasibility studies and possible outcomes Adapted from Bowen et al. [24]

The study findings are discussed under the themes of the Bowen feasibility framework [24], i.e. demand, acceptability, practicality, and integration (Fig. 2).

### Demand

Respondents felt that the provision of intramuscular artesunate as pre-referral but not definitive treatment for severe malaria at health posts could be lifesaving. Intramuscular artesunate has been shown to be highly effective in the management of severe malaria [20–22, 25–28] and WHO recommends the use of intramuscular artesunate where possible as pre-referral treatment. This WHO recommendation together with the study findings would effectively support the use of intra-muscular artesunate as pre-referral treatment at the health post, however, it was clear that this can only be possible with adequate training and with supporting evidence of safety and feasibility. Although evidence from trials of neonates with severe bacterial infection demonstrate that where referral is not possible, care provided near home can be life-saving [28], respondents in this study were not in agreement with health extension workers using intramuscular artesunate as definitive treatment citing the lack of knowledge to manage severe malaria complications as a limitation to this.

### Acceptability

The health workers in this study were aware that the national malaria treatment guidelines recommend the use of intramuscular artesunate as definitive treatment at health centres and hospitals. However, many of the study participants were not aware of the WHO recommendation to use intramuscular artesunate and rectal artesunate as pre-referral treatment even when the use of rectal artesunate as a pre-referral treatment is in the policy guidelines for Ethiopia.

The study also found that healthcare workers find it easier to administer injectable artesunate via the intramuscular route compared to the intravenous route. Given the need to find a vein and use a cannula to establish a line, it is clear why the healthcare workers find using the intramuscular route easier. Jin and others [29] report findings from a study comparing preference of administering different medication using different routes; looking at the intramuscular route versus the intravenous route and conclude that the preference varies from drug to drug. On the other hand, Ntuku and colleagues [30] in a study conducted in the Democratic Republic of Congo report that healthcare workers find use of intramuscular artesunate easier to administer than quinine which is similar to these study findings although no comparison was made with other drugs.

Lack of capacity to manage complications of severe malaria cases, and fear of irrational use of the drug were cited as reasons for not supporting the use of intramuscular artesunate as definitive treatment of severe malaria at the health post level. Concerns that health extension workers might use injectable artesunate to manage uncomplicated malaria or administer suboptimal doses are fears that need to be further explored. Little work has been done on the safety of injections provided by lay health workers [31]; most of the work is heavy on lay health workers providing injectable contraceptives and shows they can be safely delivered [32, 33]. It was recommended that operational research to further explore the safety, feasibility and efficacy of using intramuscular artesunate as definitive and pre-referral treatment be conducted to support use of intramuscular artesunate to manage severe malaria cases at the health post level especially in situations when access to higher level care is difficult or impossible. These studies will provide evidence for policy recommendations on the use of injectable artesunate at the health post level which is currently not supported by both the WHO and the Ethiopia policy guidelines.

### Practicality

The study findings indicate that the respondents felt that using intramuscular artesunate as definitive treatment at the health-post may not be possible as management of severe malaria is beyond just providing the antimalarial, but also included management of complications which the health extension workers may not be in position to do. Although the proximity of the health post to the community, and the relative ease of administration of intramuscular artesunate were stated as points that may make it a potential option to be explored. Respondents noted that introduction of intramuscular artesunate at the health post could be hindered by inadequate and unskilled health workers, lack of equipped facilities, and lack of knowledge among health post workers to manage complications resulting from severe malaria. Indeed, many of the respondents stated that with adequate training, health centre can provide definitive treatment for severe malaria using intramuscular artesunate, however, they suggested that health posts should only provide pre-referral intramuscular artesunate and not definitive treatment. The Ethiopia national malaria policy provides for definitive management of severe malaria using intramuscular artesunate at the health centre and hospital level but not the health post.

Many health workers believe the intravenous route provides flexibility to administer other injectable drugs for comorbidities, is less painful, and is convenient to the patient. Moreover, because of previous experience using the intravenous route to deliver quinine health workers maybe more inclined to favour intravenous over intramuscular delivery. Most respondents felt that the intramuscular route was easier to use than the intravenous route due to problems inserting the cannula. Health extension workers are unlikely to have the skill to use intravenous drugs but can be trained to use intramuscular drugs. Jin and others [29] argue that if the safety and efficacy of the two injection routes are equivalent, clinicians should pay attention to patient preference and pharmacoeconomics because patient preference will ensure optimal treatment adherence and ultimately improve patient experience or satisfaction, while pharmacoeconomic concern will help alleviate nurse shortages and reduce overall health care costs. Given Ethiopian’s health services are highly resources constrained (in terms of supplies, personnel, space and other resources), the intramuscular route when possible provides a cost efficient option from a public health point of view that may contribute to more equitable access to care although this was not extensively explored as part of this study.

Pre-referral intramuscular artesunate at health post level will require evidence on safety and feasibility before policy shift.

### Integration

Integration is the extent to which an idea, programme, process or measure can be incorporated within an existing system. The study findings indicate that injectable artesunate is already widely in use at hospitals and some health centres, with intravenous being the most commonly used route. From an integration perspective, in order to clarify for health workers when to use the intravenous or intramuscular route a standard operating procedure should be developed on the use of injectable artesunate.

Antero-lateral aspect of the thigh muscle is the preferred site of intramuscular administration of artesunate [6], yet other sites which are no longer recommended were mentioned by most of the respondents. This indicates the need for capacity building to ensure health workers know and use preferred safe sites for intramuscular administration.

Study limitations may include: (1) health facilities were selected purposively based on accessibility during the study period. It is possible that respondents from these facilities might have different views compared to those from areas that were inaccessible; (2) the interview guide was structured with questions on use of intramuscular artesunate by health extension workers before those related to use at the health centre level. This might have negatively influenced the way some respondents answered questions related to use of intramuscular artesunate at the health centre level, if they held the view that health extension workers should not use injectable artesunate; (3) as in most qualitative studies, the results of this study may not be generalizable but may be transferable to similar settings; (4) translation from local languages to Amharic and then to English may have affected interpretation of the results to some extent.

## Conclusion

Intramuscular artesunate is easier to administer, is widely used at hospitals, and is already in the national policy for use as definitive treatment at the health centre and hospital level in Ethiopia. From the perspective of health workers in this study, use of intramuscular artesunate as pre-referral treatment of severe malaria cases at the health post is possible but dependent on training and availability of skilled workers. Use of intramuscular artesunate as definitive treatment at health posts was not supported due to lack of skills by the health extension workers to manage complications associated with severe malaria, and operational research to establish its feasibility, safety and efficacy of use of injectable artesunate as pre-referral and definitive treatments at the health post was recommended to guide any implementation of such an intervention.

## Abbreviations

WHO: World Health Organization
SNNPR: southern nations, nationalities, and peoples’ region
